# Supplementary material for: A Closer Look at Dexamethasone and the SARS-CoV-2-Induced Cytokine Storm: In Silico Insights of the First Life-Saving COVID-19 Drug
Source: Antibiotics (Basel). 2021 Dec 8;10(12):1507. doi: 10.3390/antibiotics10121507 (PMC8698520; doi:10.3390/antibiotics10121507)
Supplement: Supplementary file 1 [file antibiotics-10-01507-s001.zip › antibiotics-1462208-supplementary.pdf]

Supplementary Materials

Figure S1 a-t. Shows the 2D ligand interaction with dexamethasone and screened targets.

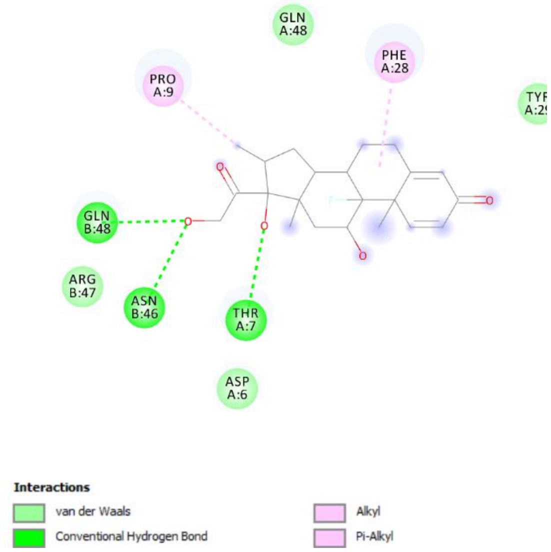

a. CCL5 and Dexamethasone 2D interaction diagram

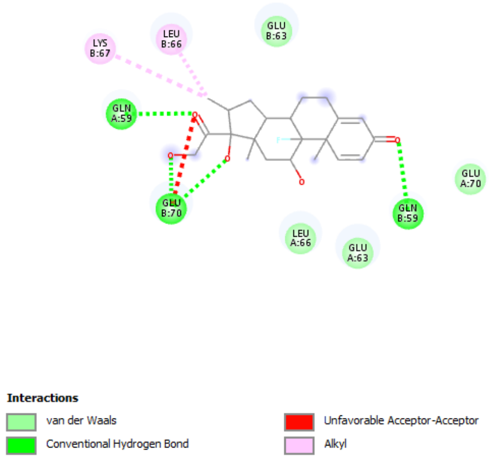

b. CXCL8 and Dexamethasone 2D interaction diagram

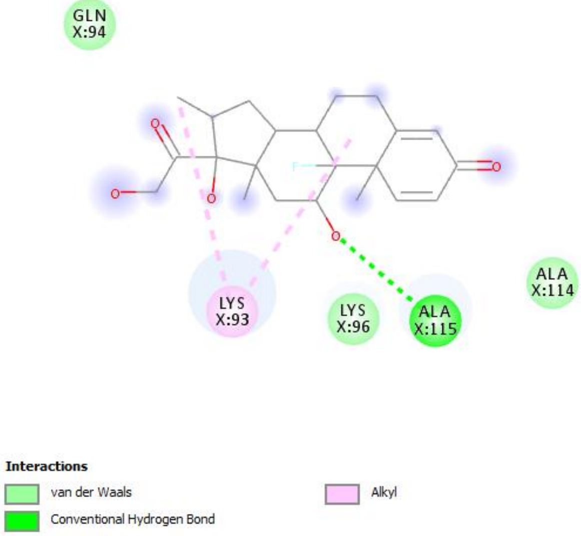

c. IL-1 Dexamethasone 2D interaction diagram

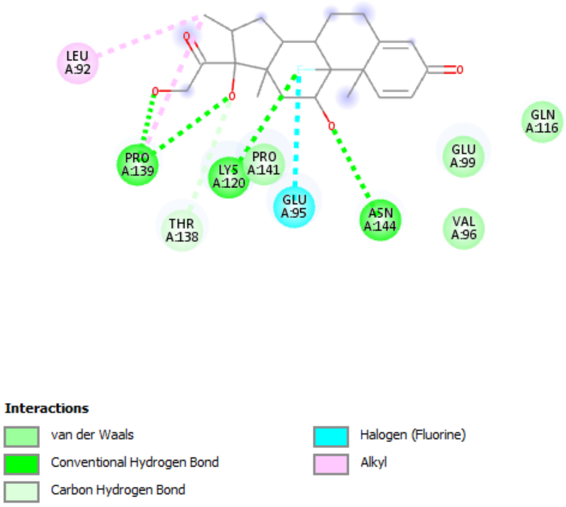

d. IL-6 Figure 4 and Dexamethasone 2D interaction diagram

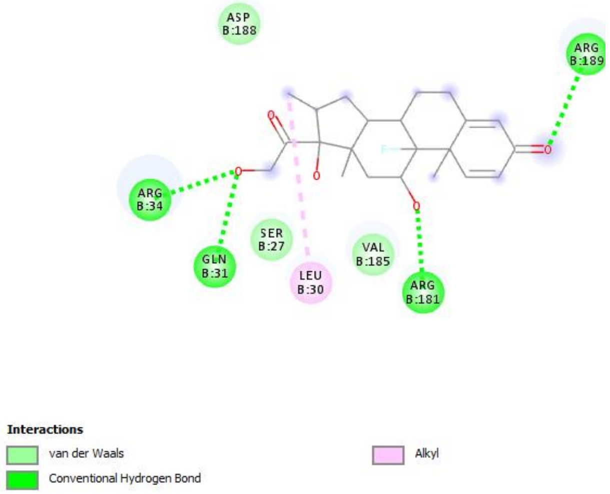

e. IL-12 Dexamethasone 2D interaction diagram

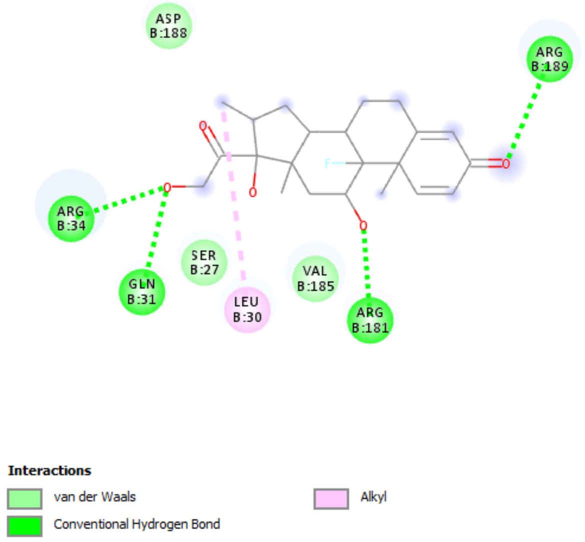

f. IL-12 Dexamethasone 2D interaction diagram

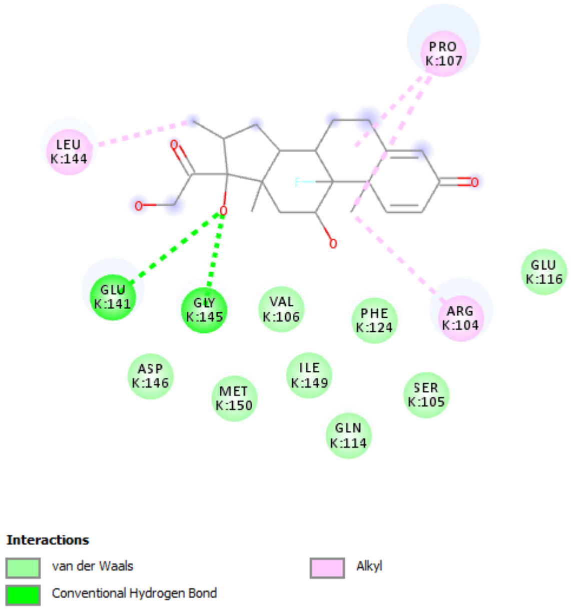

g. IL-18 and Dexamethasone 2D interaction diagram

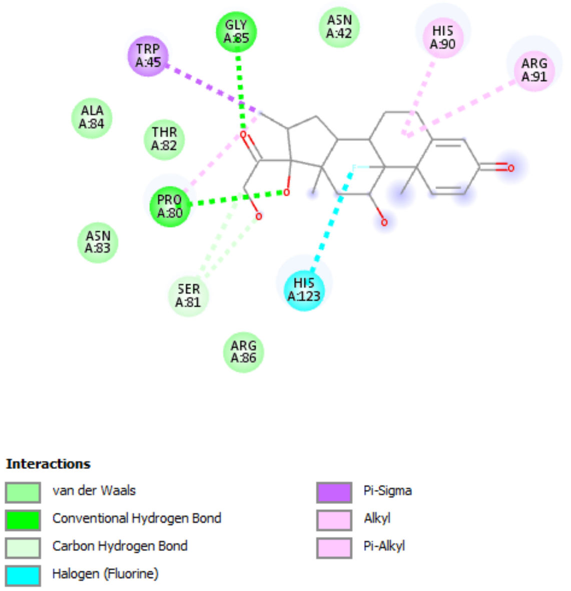

h. IL-21 and Dexamethasone 2D interaction diagram

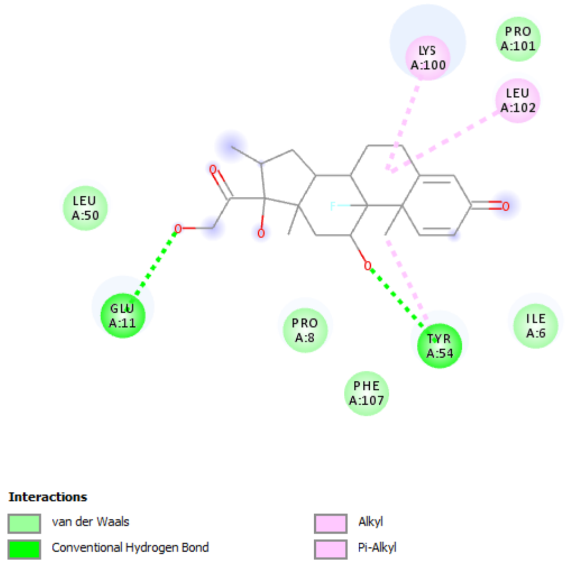

i. IL-33 and Dexamethasone 2D interaction diagram

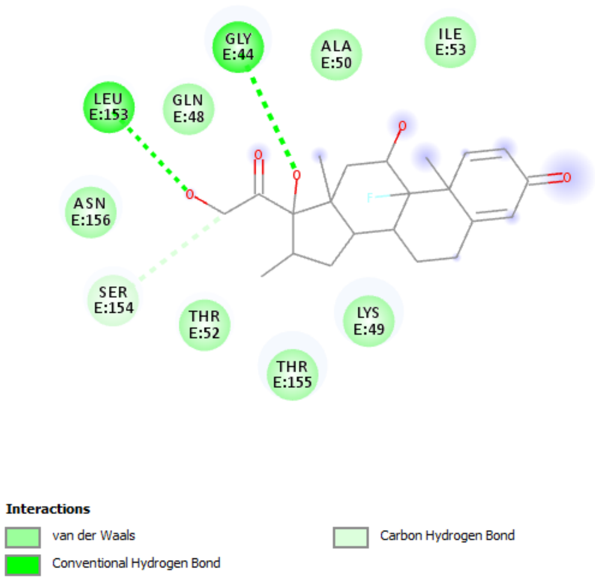

j. S10 INFa2 and Dexamthasone 2D interaction diagram

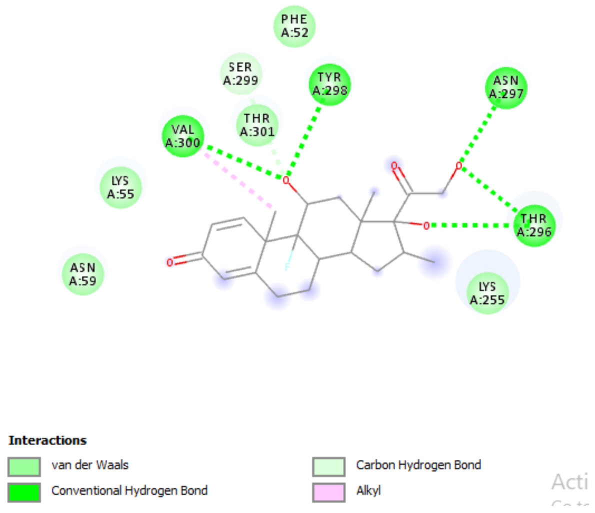

k. INFg and Dexammethasone 2D interaction diagram

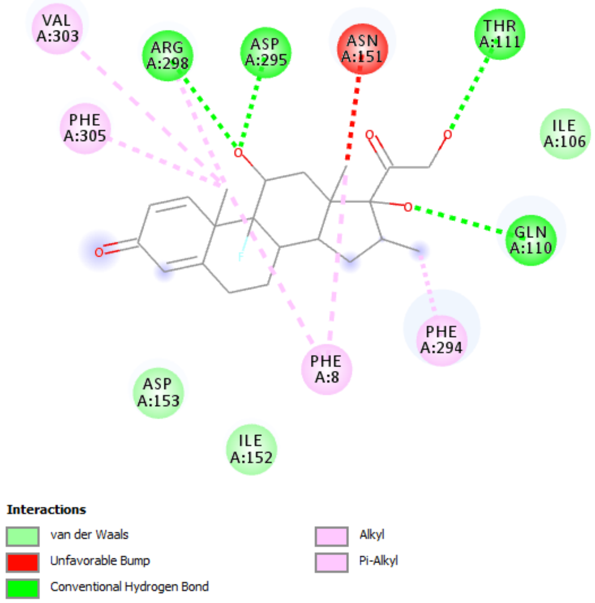

l. S12 Mpro and Dexamethasone 2D interaction diagram

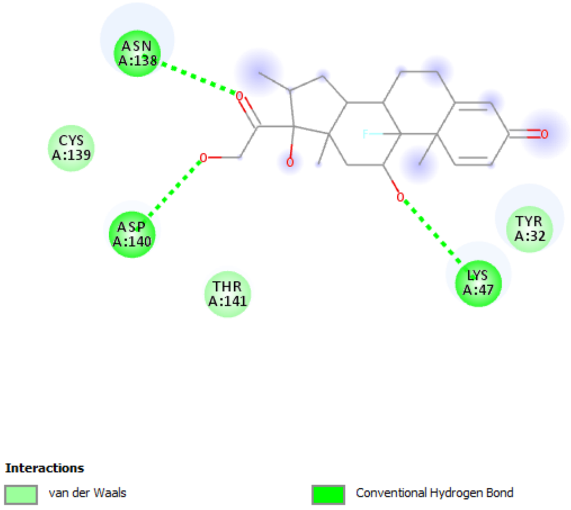

m. RNA Polymerase and Dexamethasone 2D interaction diagram

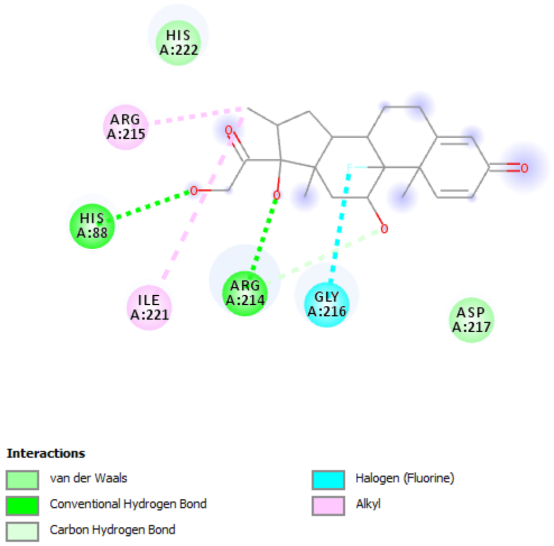

n. TGFB1 and Dexamethasone 2D interaction diagram

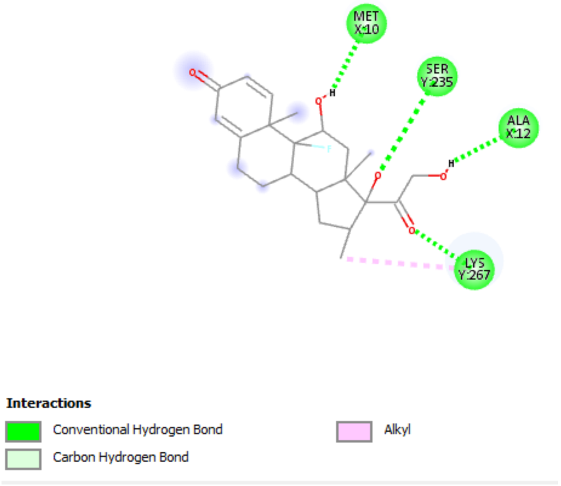

o. IL-1 Receptor and Dexamethasone 2D interaction diagram

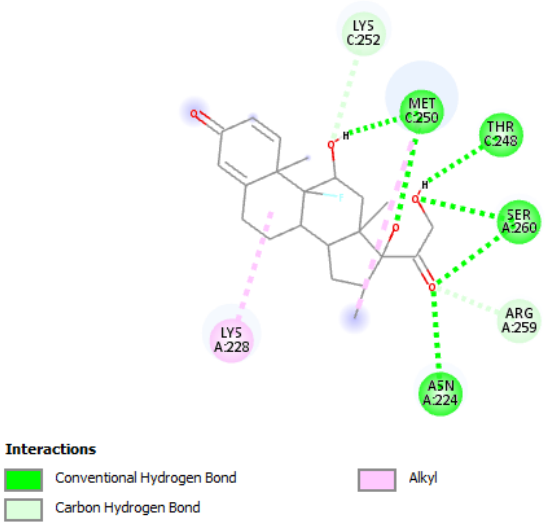

p. IL-6 Receptor and Dexamethasone 2D interaction diagram

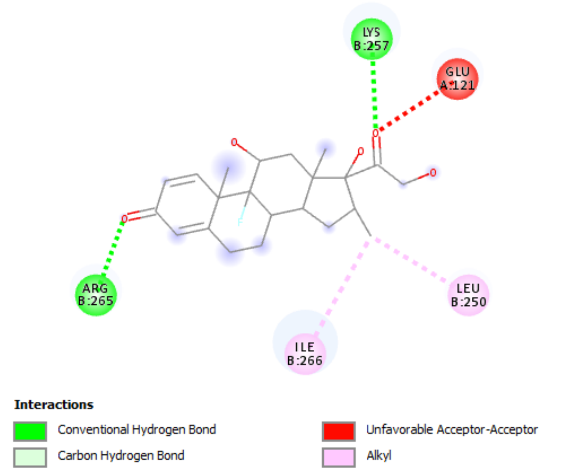

q. IL-33 Receptor Receptor and Dexamethasone 2D interaction diagram

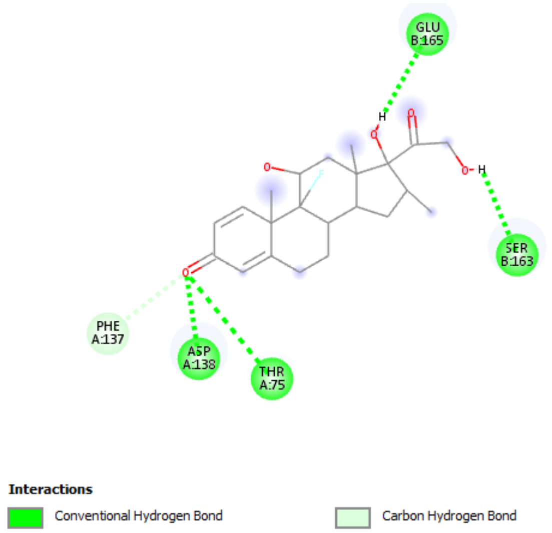

r. INFA2 Receptor and Dexamethasone 2D interaction diagram

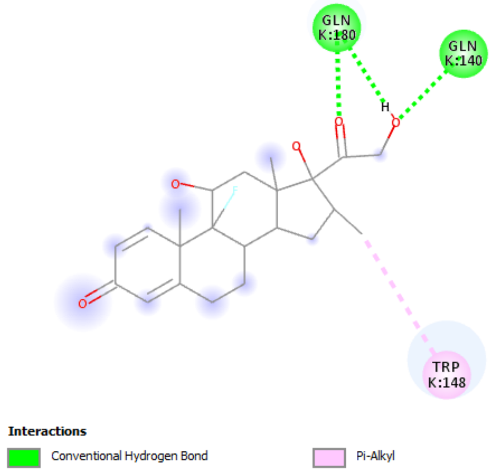

s. IL-21 Receptor and Dexamethasone 2D interaction diagram.

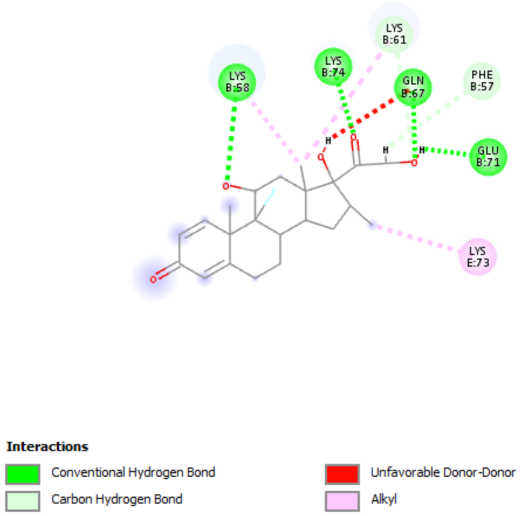

t. IFNG Receptor and Dexamethasone 2D interaction diagram.

Table S1. Shows the molecular weight (kDa) of the cytokines and chemokines that were emoplyed in the study. An median molecular weight of 17.3 kDa demonstrates that cytokines and chemokines are realtievely small in the currently know protien universe.

Inflammatory Marker (IM)	PDB Code	Molecular Weight (kDa)
**IFNa1**	3UX9	22
**IL-1B**	5MVZ	17.5
**IL-12**	1F45	57.89
**IL-18**	3WO3	7.2
**IL-33**	2KLL	18.16
**INFa2**	4Z5R	19
**INFg**	1FYH	17
**TGFB1**	5vqp	25
**TGFB2**	4KXZ	12.7
**TGFB3**	1TGK	47.2
**TNF**	1TNF	17.3
**IL-1a**	1IRA	31
**IL-6**	1ALU	21
**IL-8**	5D14	8.4
**IL-21**	2OQP	15.4
**CCL1**	1DOK	15.5
**CCL2**	2LIE	13
**CCL3**	3FPU	7.8
**CCL**	1RTN	15.7
**CXCL8**	1ILQ	18.87
**CXCL 10**	1O80	10
	Mean:	**19.89**
	Median:	**17.30**
